# Supercritical Fluid Extraction and Ultra Performance Liquid Chromatography of Respiratory Quinones for Microbial Community Analysis in Environmental and Biological Samples

**DOI:** 10.3390/molecules17032628

**Published:** 2012-03-05

**Authors:** Muhammad Hanif, Yoichi Atsuta, Koichi Fujie, Hiroyuki Daimon

**Affiliations:** 1 Department of Environmental and Life Sciences, Toyohashi University of Technology, Aichi 441-8580, Japan; Email: hanif@water.ens.tut.ac.jp (M.H.); atsuta@water.ens.tut.ac.jp (Y.A.); 2 Center for Energy Resources Development, Agency for the Assessment and Application of Technology, Jakarta 10340, Indonesia; 3 Graduate School of Environment and Information Sciences, Yokohama National University, Kanagawa 240-8501, Japan; Email: fujie@ynu.ac.jp

**Keywords:** biological sample, environmental sample, liquid chromatography menaquinone, quinone profile, supercritical fluid extraction, ubiquinone

## Abstract

Microbial community structure plays a significant role in environmental assessment and animal health management. The development of a superior analytical strategy for the characterization of microbial community structure is an ongoing challenge. In this study, we developed an effective supercritical fluid extraction (SFE) and ultra performance liquid chromatography (UPLC) method for the analysis of bacterial respiratory quinones (RQ) in environmental and biological samples. RQ profile analysis is one of the most widely used culture-independent tools for characterizing microbial community structure. A UPLC equipped with a photo diode array (PDA) detector was successfully applied to the simultaneous determination of ubiquinones (UQ) and menaquinones (MK) without tedious pretreatment. Supercritical carbon dioxide (scCO_2_) extraction with the solid-phase cartridge trap proved to be a more effective and rapid method for extracting respiratory quinones, compared to a conventional organic solvent extraction method. This methodology leads to a successful analytical procedure that involves a significant reduction in the complexity and sample preparation time. Application of the optimized methodology to characterize microbial communities based on the RQ profile was demonstrated for a variety of environmental samples (activated sludge, digested sludge, and compost) and biological samples (swine and Japanese quail feces).

## 1. Introduction

Currently, there is a growing interest in the application of microbial community structure in many areas of study. Knowledge of microbial community structure has been used to optimize the performance of wastewater treatment, composting, and anaerobic digestion processes [1,2,3,4]. Moreover, in animal husbandry and poultry industries, the analysis of microbial communities in animal feces also plays an important role in the enhancement of animal health and productivity as well as the performance of fecal pollution detection methods [5,6]. Although several methods have been utilized for determining the microbial community structure in organic samples, the development of a superior analytical strategy for solid environmental and biological samples is still necessary.

Analysis of respiratory quinone (RQ) composition is one of the most widely used culture-independent techniques for characterizing microbial community structure [7,8,9,10] and has provided new insights into the structure of the microbial communities and their abundance. The structure and diversity of microbial communities can be indicated by the quinone composition because most microorganisms contain a major quinone species [11,12]. The quinone profile is usually represented as the mole fraction of each quinone type. Changes in a microbial community in a mixed culture of microbes could effectively be quantified using the quinone profile.

**Figure 1:**
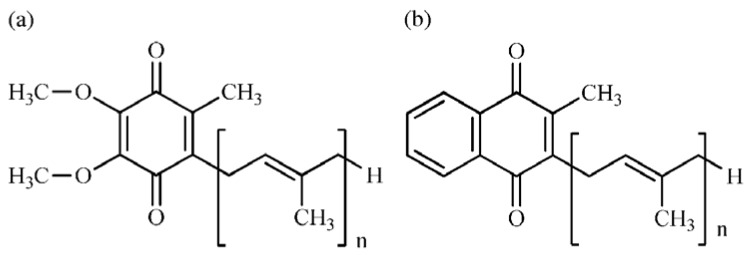
Chemical structures of (**a**) ubiquinone (UQ-n (Hx)) and (**b**) menaquinone (MK-n (Hx)), where n is the length of the isoprene unit of the side chain and x is the number of hydrogens atoms saturating the double bonds of the isoprene unit.

Quinones are lipid-soluble substances that are widely distributed in nature [13,14] and in almost all species of organisms [11,15]. They are classified into two major groups: ubiquinones (1-methyl-2-isoprenyl-3,4-dimethoxyparabenzoquinone) and menaquinones (1-isoprenyl-2-methyl-naphtho-quinone). Ubiquinones (UQ, coenzyme Q) and menaquinones (MK, vitamin K2) occur most frequently in bacteria and are in the bacterial plasma membrane where they participate in electron transport [13,15]. The chemical structures of ubiquinone and menaquinone are shown in Figure 1. Both UQ and MK have polyprenyl side chains which vary in the number of isoprene units and in the degree of unsaturation. This structural variation in the polyprenyl side chain is the basis for bacterial classification [11,15].

In the conventional method, analysis of quinones is performed by the organic solvent extraction (direct extraction) with chloroform-methanol mixture [7,9,11]. However, the conventional method has still some disadvantages: Time-consuming, tedious procedures and the use of large volume of toxic solvents.

One of the new strategies for determining microbial community structure is the application of supercritical fluid extraction (SFE). SFE is a green technology that offers numerous advantages during analysis, mainly rapidity, selectivity and low solvent volume usage [16,17]. In addition, increased awareness of the environmental, health and safety issues in the use of hydrocarbon solvents has also increased the studies of SFE.

We previously found that a modified supercritical carbon dioxide (scCO_2_) extraction with methanol rivaled the conventional organic solvent extraction methods for the characterization of bacterial RQ in activated sludge [18,19]. The solvent collection or solid-phase collection method was not applied as trapping media in our previous scCO_2_ extraction method. Thus, an empty vessel was used to collect the extracted RQ in SFE system. Tedious purification and separation steps were required to obtain high recovery of RQ from each sample. The type and concentrations of UQ and MK were determined separately by a high performance liquid chromatography (HPLC) analysis. The extraction and analysis time of one sample can take more than 6 h. The application of this method to determine bacterial RQ from various environmental samples other than activated sludge such as compost and soil showed a lower yield than conventional organic solvent extraction. HPLC has been commonly used for the determination of microbial quinones after extraction from the sample. In other studies, liquid chromatography-atmospheric pressure chemical ionization mass spectrometry (LC-APCI-MS) has also been used for the identification of UQ and MK from environmental samples [20,21].

Therefore, the objective of the present work is to develop a more effective RQ analysis method by directly mounting the solid-phase extraction cartridge in-line on the SFE instrument. The use of a solid-phase cartridge has the potential to trap bacterial RQ from the samples in one step without further purification. The applicability of the method for the analysis of bacterial RQ from a wide variety of samples (environmental and biological) was investigated. Activated sludge, digested sludge and compost were used as environmental samples. Swine feces and Japanese quail feces were used as biological samples. The conventional organic solvent extraction method was used to evaluate the reliability of the SFE with solid-phase trapping method. The ultra-performance liquid chromatography (UPLC) was also introduced to analyze bacterial UQ and MK simultaneously. The UPLC is relatively new technique in liquid chromatography giving new possibilities to identify bacterial RQ. This technique could provide a higher peak capacity, a greater resolution and sensitivity as well as a high speed analysis over conventional HPLC [22,23].

## 2. Results and Discussion

### 2.1. Comparison of HPLC and UPLC on the Analysis of UQs and MKs

Previous studies on microbial quinone extraction with scCO_2_ have shown that a significant amount of quinones could be extracted from the activated sludge sample at a temperature of 55 °C, a pressure of 25 MPa, and 10% (v/v) methanol for 15 min [18,19]. At these conditions, the quinone contents obtained by scCO_2_ extraction using methanol as a modifier were compared to those obtained by organic solvent extraction. Table 1 shows the comparison of HPLC and UPLC on the analysis of UQs and MKs in the anaerobically digested sludge. The total extracted quinones analyzed with HPLC and UPLC were 250.15 ± 3.51 and 263.19 ± 9.50 µmol/kg-dry sludge, respectively. However, UPLC has been performed almost twice as quickly as HPLC. The results also showed that the same amount of quinones were obtained from UPLC without quinone fractionation, suggesting that the separation and purification procedure with a Sep-Pak cartridge could be eliminated which subsequently reducing the analytical time and solvent consumption.

**Table 1:** The comparison of HPLC and UPLC for the analysis of UQs and MKs in the anaerobically digested sludge (mean ± SD, n = 3).

Quinone Homolog		HPLC (µmol/kg-dry)		UPLC (µmol/kg-dry)		
		Fractionated		Fractionation		Un-fractionated
UQ-7		0.00 ± 0.00		4.45 ± 0.47		5.94 ± 0.58
UQ-8		54.10 ± 4.78		43.60 ± 2.36		44.14 ± 3.71
UQ-9		27.25 ± 1.72		28.89 ± 2.32		30.63 ± 2.12
UQ-10		83.99 ± 2.62		71.24 ± 4.63		70.49 ± 2.13
MK-6		11.27 ± 0.43		10.25 ± 0.38		8.86 ± 0.19
MK-7		26.71 ± 2.00		49.55 ± 2.35		46.03 ± 3.30
MK-8		7.03 ± 0.33		7.39 ± 0.15		8.19 ± 0.55
MK-9		7.55 ± 2.04		9.31 ± 0.35		10.23 ± 0.28
MK-9(H_2_)		3.43 ± 0.09		4.32 ± 0.19		5.26 ± 0.29
MK-8(H_4_)		5.26 ± 0.97		7.13 ± 0.59		8.87 ± 0.22
MK-9(H_4_)		3.10 ± 1.21		3.53 ± 0.72		3.28 ± 1.07
MK-10(H_4_)		20.43 ± 2.35		22.80 ± 0.96		21.30 ± 0.48
Total		250.15 ± 3.51		262.55 ± 12.69		263.19 ± 9.50

The effectiveness of HPLC and UPLC methods is comparable to each other based on the values of dissimilarity index. The dissimilarity index was calculated by the following equation [24,25,26]: 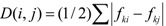, where 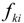 and 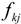 are molar fractions of a quinones species *k* in communities *i* and *j*, respectively. Both analysis methods could be considered different when the dissimilarity index is greater than or equal to 0.1 [24]. Compared to the HPLC method, the values of the dissimilarity index of the quinone profiles by the UPLC methods were 0.04 and 0.07, respectively. These values mean that the quinone profiles of HPLC and UPLC methods were similar. 

### 2.2. Optimization of the SFE Methods

**Figure 2:**
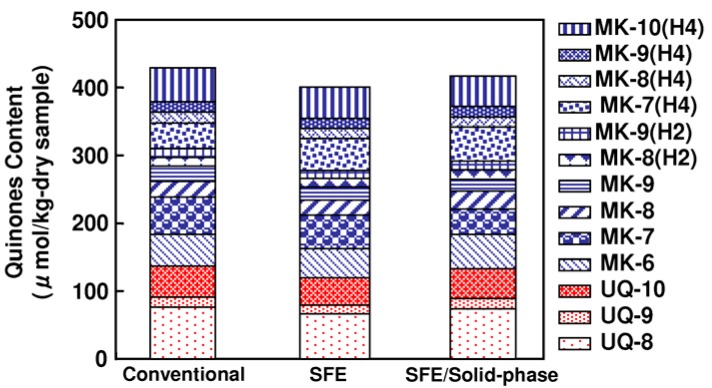
Effect of different treatments on the total content of the extracted quinones from activated sludge; conventional solvent extraction method (Conventional); SFE/UPLC off-line with Sep-Pak cartridge (SFE); SFE/UPLC in-line with Sep-Pak cartridge (SFE/Solid-phase) (n = 3).

The effect of different SFE and UPLC treatment methods on the total content of extracted quinones from activated sludge is shown in Figure 2. Three different treatments were performed to find the most effective method of producing a high yield of extracted quinones. The extraction of the quinones from the samples was initially performed using a conventional organic solvent extraction method. Then, a method was developed using scCO_2_ extraction off-line with Sep-Pak cartridge separation and purification and finally, a scCO_2_ extraction in-line with Sep-Pak cartridge.

The conventional method was able to extract quinones from activated sludge with relatively high yield (420 ± 16.34 µmol/kg-dry sample) compared to the SFE methods. Thirteen quinone species were identified in activated sludge samples; nevertheless, it is well known that this method has many drawbacks, including the fact that it is time-consuming and uses a large volume of carcinogenic solvent.

The total content of the extracted quinones from the activated sludge samples was determined to be 390 ± 12.44 and 410 ± 18.62 µmol/kg-dry by the off-line scCO_2_ extraction with Sep-Pak cartridge method and by the in-line scCO_2_ extraction with Sep-Pak cartridge method, respectively. The total quinone content extracted by scCO_2_ was comparable to that obtained with the conventional method. Methanol was used as a trapping solvent for the scCO_2_ extraction off-line with Sep-Pak cartridge method.

The value of the dissimilarity index was also used to compare the methods. Compared to the conventional method, the values of the dissimilarity index of the quinone profiles by the SFE method were 0.03 and 0.05, respectively. This value means that both profiles of the quinones were similar. Both quinone profiles could be considered different when the dissimilarity index is greater than or equal to 0.1 [24].

These findings demonstrated the possibility of extracting ubiquinones and menaquinones from activated sludge with scCO_2_ extraction with offline or line Sep-Pak cartridge. It should be noted that impurities, such as water-soluble materials from extracts, might be obtained during the analysis.

**Figure 3:**
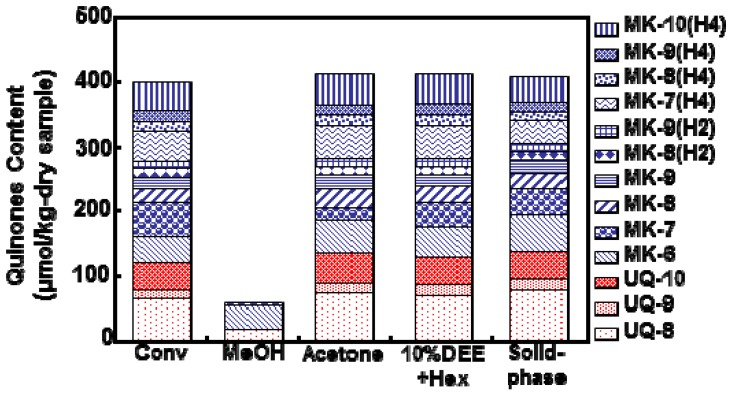
Effect of trapping system on the total content of the extracted quinones; conventional method (Conv); solvent trapping using methanol (MeOH); solvent trapping using acetone (acetone); solvent trapping using a mixture of 10% diethyl ether and hexane (10% DEE/Hex); solid trapping using Sep-Pak cartridge (solid-phase) (n = 3).

Figure 3 shows the effect of an SFE trapping system on the total content of the extracted quinones from activated sludge compared to the conventional method. Four different SFE trapping procedures were performed: solvent trapping using methanol, acetone, a mixture of 10% diethyl ether and hexane, and a solid trapping using Sep-Pak cartridge. These different trapping methods were compared to conventional organic solvent extraction method.

The SFE experiments using acetone trapping, a mixture of 10% diethyl ether and hexane trapping, and Sep-Pak cartridge trapping were comparable to those obtained with the conventional method (420 ± 16.34 μmol/kg-dry sample). Comparatively, experiments using methanol trapping gave a lower content of the total quinones extracted. Considering the time saved and the ease of the procedure, SFE combined directly with Sep-Pak cartridge trapping became the selected method to extract quinones from 100 mg samples under the tested conditions.

### 2.3. Validity of the Developed Method

To validate the SFE and UPLC method, standard spiking experiments were performed. Only UQ-10 and MK-7 were used as standard solution because other quinone standards were not commercially available. Standard solutions were prepared by diluting the stock solutions in acetone. The clean glass wool was placed to fill the void volume of the extraction vessel. Then, standard solution of 0.5 mL was added. The extraction vessel was capped and placed into the SFE system immediately. SFE was then performed at 25 MPa, 55 °C for 15 min.

Triplicate experiments were conducted to determine the level of precision. Three concentrations were used, and three replicates of analysis on the same sample were performed, and expressed in terms of the relative standard deviation (RSD).

The UPLC method used in this study was developed to obtain good peak resolution and a high speed of analysis. The UPLC method was validated in terms of precision, accuracy and linearity, limit of detection (LOD) and limit of quantification (LOQ) [27]. The precision was evaluated by means of repeatability measures. The accuracy of the developed method was defined as the difference between the experimental results and the true value. The linearity was measured by performing triplicate injections of the standard solution at 1, 10, 25, 50, 100, 250, 725 and 1,000 µg/L. Calibration curves were plotted based on the peak area and concentrations. The curves were linear over the calibration range at the correlation coefficient of *R*^2^ > 0.99. The LOD was determined as the concentration with a signal to noise ratio (S/N) of 3. The LOQ was defined as the lowest validated level meeting the requirements of a recovery within an RSD < 20%. The mean ± SD percent recoveries of UQ-10 and MK-7 were 97 ± 5 and 98 ± 7, respectively. The RSD for the triplicate analysis of spiked samples ranged from 5% to 14%. The results indicated that the UPLC equipped with a photo diode detector performed acceptably despite interference by the sample matrices. Under the UPLC conditions, the LODs achieved by spiking experiments were estimated to be 5 and 60 µg/kg for UQ-10 and MK-7, respectively. The LOQ values were calculated to be 10 and 120 µg/kg for UQ-10 and MK-7, respectively.

**Figure 4:**
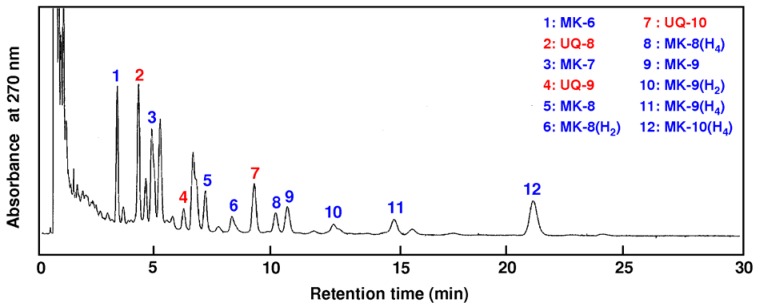
UPLC chromatograms of ubiquinones (UQ) and menaquinones (MK) extracted from anaerobically digested sludge under scCO_2_ extraction in-line with solid phase cartridge method.

Figure 4 shows the chromatograms of the ubiquinones and menaquinones extracted from digested sludge using scCO_2_ extraction in-line with solid phase cartridge at a temperature of 55 °C, a pressure of 25 MPa, a CO_2_ flow rate of 2.7 mL/min and a methanol flow-rate of 0.3 mL/min for 15 min. Liquid chromatography methods were performed at 35 ± 1 °C, the mobile phase consisted of 97% methanol containing 3% v/v di-isopropyl ether, and the flow rate was 0.5 mL/min with a chromatographic run time of 35 min. Three ubiquinone species (UQ-8, UQ-9 and UQ-10) and 9 menaquinone species (MK-6, MK-7, MK-8, MK-8(H_2_), MK-8(H_4_), MK-9, MK-9(H_2_), MK-9(H_4_) and MK-10(H_4_)) were identified by the retention time and the spectrum. These results demonstrated the possibility of extracting ubiquinones and menaquinones from the anaerobically digested sludge by scCO_2_ extraction and UPLC analysis. The UPLC technique proved to be more sensitive and rapid to determine bacterial RQ compare to conventional HPLC.

### 2.4. Comparison of Supercritical CO_2_ Extraction with Organic Solvent Extraction for Various Environmental and Biological Samples

**Figure 5:**
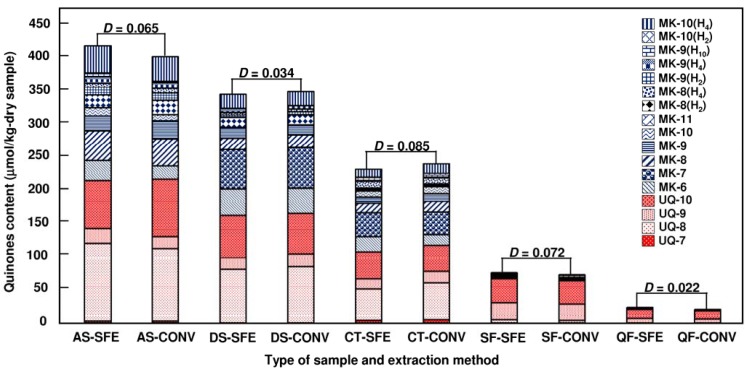
Comparison of the quinone compositions by supercritical fluid extraction (SFE) and an conventional organic solvent extraction (CONV) method in environmental and biological samples; activated sludge (AS); digested sludge (DS); compost (CT); swine feces (SF); quail feces (QF). The *D* values represent the dissimilarity index; the data show the average value of three different determinations for each sample.

The applicability of the method was tested for different environmental and biological samples. Optimized supercritical CO_2_ extraction conditions from previous experiments were applied: an extraction vessel temperature of 55 °C, a pressure of 25 MPa, a CO_2_ flow rate of 2.7 mL/min, and a methanol flow rate of 0.3 mL/min for 15 min. The composition and structure of the quinone species from the different environmental and biological samples are shown in Figure 5. The quinones that were identified in activated sludge, digested sludge, compost, swine fecal and Japanese quail fecal correspond with those suggested previously as typical biomarkers for the expected bacteria [11,12,13]. Among the ubiquinones and menaquinones, UQ-8, UQ-9, UQ-10 and MK-9(H_2_) were identified in almost all the samples. The other types of quinones such as UQ-7, MK-11 and MK-9(H_4_) existed in few samples. Four species of ubiquinones and 10 species of menaquinones were extracted from the activated sludge (AS) samples, three ubiquinones and nine menaquinones were extracted from the digested sludge (DS) samples, four ubiquinones and eight menaquinones were extracted from the compost (CT) samples, three ubiquinones and five menaquinones were extracted from the swine feces (SF) samples and three ubiquinones and two menaquinones were extracted from the quail feces (QF) samples. The total content of extracted quinone of the AS, DS, CT, SF and QF samples was 417.70 ± 24.56, 344.35 ± 18.23, 232.07 ± 10.97, 75.05 ± 8.79, 22.57 ± 7.85 µmol/kg dry sample, respectively.

The quinone contents obtained by scCO_2_ extraction in-line with Sep-Pak cartridge method were compared to those obtained by organic solvent extraction. The experiments for both scCO_2_ extraction and conventional organic solvent extraction were conducted thrice for each identical sample amount. The total quinone amount extracted by scCO_2_ extraction was comparable to, or slightly lower than, that obtained when using an organic solvent extraction. 

The comparison of the scCO_2_ extraction method and the conventional extraction method for each sample was expressed based on the dissimilarity index. It was found that the values of dissimilarity (*D*) between the two methods for all samples were lower than 0.1. The two quinone profiles can be considered different statistically when the dissimilarity index is greater than or equal to 0.1 [24]. The effectiveness of the extraction of both methods for all samples was similar based on the values of the dissimilarity index. Thus, the developed scCO_2_ extraction method can be used as a replacement for the conventional method. 

Total amount and composition of RQ species were varied between different samples used in this study, indicating the degree of variability in bacterial community. Physical, chemical, and biological factors (*i.e.*, sample material type and texture, sample treatment, carbon source, moisture, pH, temperature) are believed to affect bacterial community composition. These factors interact with each other and have both direct and indirect effects on the bacterial community. 

Activated sludge used in this study was a typical mixed culture of microorganisms from the aeration tank of a domestic wastewater treatment plant. The results showed that UQ-8, UQ-10, MK-8, MK-10(H_4_), MK-9(H_2_) and MK-6 were the predominant bacterial RQ in the activated sludge. These RQ species indicated alpha-, beta-, and gamma-proteobacteria and actinobacteria as typical bacterial community in activated sludge [11]. The same results were also obtained in our previous studies with the total amount of RQ of 507 µmol/kg-dry sample [18]. Anaerobically digested sludge used in this study was the semi-solid material consisting of a complex mixture of microbial fragments and organic colloids released during anaerobic digestion of food waste and cow manure. The predominant bacterial RQ in digested sludge were UQ-8, UQ-10, MK-6 and MK-7, indicating gram positive bacteria, delta-proteobacteria and actinobacteria. Delta-proteobacteria indicates possible presence of sulfur-reducing bacteria in anaerobic process [2]. Composting is the biological decomposition of organic matter under controlled aerobic conditions [4]. Compost samples used in this study derived from waste wood and cow manure with UQ-8, UQ-10 and MK-6 as typical major bacterial RQ. The animal fecal samples from swine and quail feces used in this study as biological samples were collected from dried solid feces of healthy swine and quail. The samples consisted of organic matter and plant nutrients with only UQ-9 and UQ-10 appeared as the major bacterial RQ. The plant nutrients in animal feces are mostly present in the inorganic form, while the organic matters are derived from undigested feed and bacteria in the feces.

## 3. Experimental

### 3.1. Standard Solutions and Reagents

The ubiquinone standard (UQ-10) and the menaquinone standard (MK-7) were purchased from Nacalai Tesque (Kyoto, Japan). Standard stock solutions of 1,000 mg/L were prepared in acetone. These solutions were stored at 4 °C. Acetone, methanol, di-isopropyl ether, and hexane were HPLC grade (Wako Co., Osaka, Japan). Two kinds of solid-phase cartridges were used in this study. In conventional and SFE off-line method, the Sep-Pak Plus® Silica cartridges (2 × 1 cm I.D.; polypropylene tubes; silica gel content: 600 mg; particle diameter: 55–105 μm) purchased from Waters (Milford, MA, USA) were used for sample preparation. In SFE in-line, Sep-Pak^®^ Plus C_18_ silica cartridges (polypropylene tubes; sorbent weight: 360 mg; particle size: 55–105 µm; pore size: 125 Å) purchased from Waters Co., Japan, and were used as a solid-phase trapping cartridge.

### 3.2. Sample Preparation

The activated sludge (AS) samples used in this study were collected from the aeration tank of a domestic wastewater treatment plant at Toyohashi University of Technology, Aichi, Japan. The digested sludge (DS) samples were obtained from the anaerobic digestion of a mixture of cow manure and food waste at an anaerobic pilot plant of Maezawa Industries Co. Ltd., Japan. The compost (CT) samples derived from waste wood and cow manure were provided by Komatsuya Co. Ltd., Japan. The swine feces (SF) and Japanese quail feces (QF) samples used in this study were obtained from breeders in Toyohashi, Aichi, Japan. These biological samples were collected daily from healthy swine and quail.

Prior to the SFE experiments, all samples were dried in a vacuum-freeze dryer for 24 h. To obtain a specific particle size distribution for the extraction, the samples were grinded and sieved to particles smaller than 500 μm. Freeze-dried samples of AS, DS, CT, SF and QF were stored at −20 °C until analysis. A freeze-dried sample of 100 mg was used for conventional organic solvent extraction and supercritical fluid extraction (SFE).

### 3.3. Analytical Strategies for Bacterial RQ Analysis

**Scheme 1:**
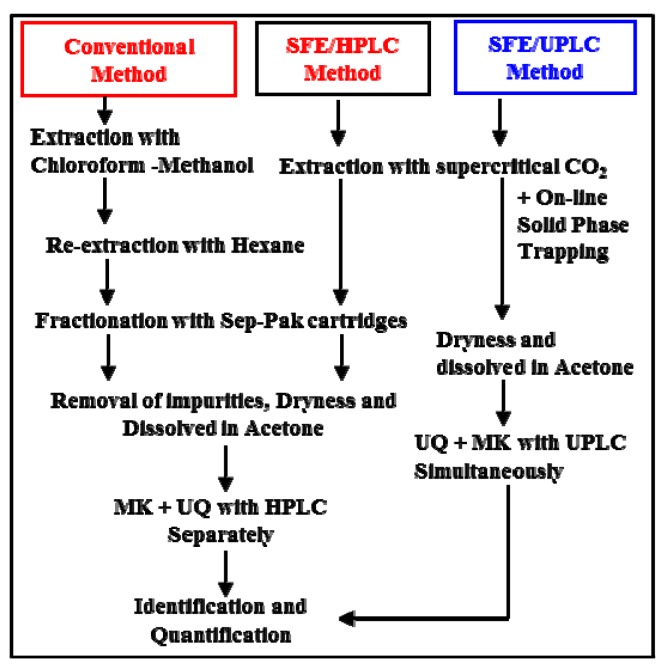
Development scheme of analytical strategies for the quinone profile method using a conventional solvent extraction method and a supercritical fluid extraction method.

The development of analytical strategies for bacterial RQ analysis using both a conventional organic solvent extraction method and SFE methods is illustrated in Scheme 1. Conventionally, analysis of quinones has been performed by organic solvent extraction with a chloroform-methanol mixture [9]. Samples containing quinones are separated into a complex mixture of lipids and impurities. The impurities are separated by a solid-phase extraction method using Sep-Pak Plus^®^ Silica cartridge [9,12]. Bacterial quinones are separated into ubiquinones and menaquinones in the solid phase extraction according to their polarities [9]. The ubiquinones and menaquinones compositions of the purified fractions are then analyzed separately by HPLC. In the off-line SFE method, some protocol steps such as extraction with hexane and purification were removed to simplify the procedure. In the in-line SFE method, the solid-phase extraction cartridge (Sep-Pak C_18_ Silica Cartridge) was mounted in-line on the SFE instrument. UPLC was used to analyze bacterial UQ and MK simultaneously.

### 3.4. Conventional Organic Solvent Extractions

The conventional method was characterized by the single-phase extraction system using a mixture of methanol and chloroform followed by washing and solid phase fractionation. Extracting twice with hexane was performed to recover quinones from the crude quinone extract. Two Sep-Pak cartridge column that were joined in series were used instead of column chromatography or thin layer chromatography (TLC) to simultaneously purify and separate quinones from the hexane extract based on their different polarities. Five milliliters of n-hexane was passed through the Sep-Pak cartridges. Then, the n-hexane extract containing the quinones was loaded onto the cartridges at a fixed flow rate of 24 mL/min. The eluted fractions from the Sep-Pak were evaporated to dryness, and then the evaporated residues were re-dissolved in acetone for future quantitative analysis by UPLC.

### 3.5. Supercritical Fluid Extractions

**Figure 6:**
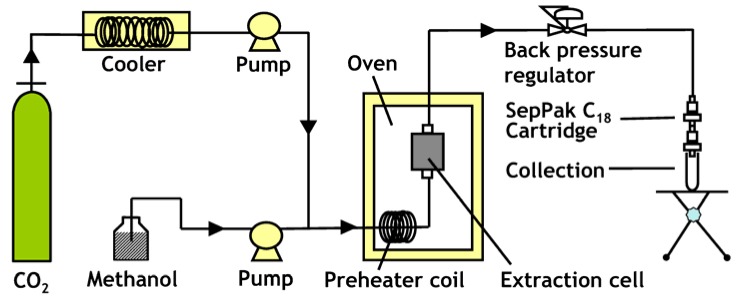
Schematic diagram of the supercritical carbon dioxide extraction system used in the present study.

All the experiments were performed using an SFE system as illustrated in Figure 6. A freeze-dried sample of 100 mg was placed in an extraction vessel (1 mL internal volume, SUS 316, 47 mm long, 10 mm I.D.) supplied by Jasco (Tokyo, Japan). CO_2_ was delivered by a Jasco PU-1580 HPLC pump (Tokyo, Japan). Methanol, used as a modifier, was delivered by a Jasco PU-2086 HPLC pump (Tokyo, Japan). A six-way switching valve was used to by-pass the extraction vessel which was placed in a GL Sciences GC-353B oven (Tokyo, Japan). The preheating coil (2 m) was used to ensure uniform delivery of a mixture of CO_2_ and methanol during the extraction process. The pressure in the system was monitored using a back pressure regulator (BPG) Jasco-BPG 880-81 (Tokyo, Japan). The BPG was heated to 55 °C to avoid dry ice formation.

The SFE conditions were as follows: an extraction vessel temperature of 55 °C, a pressure of 25 MPa, a CO_2_ flow rate of 2.7 mL/min, and a methanol flow rate of 0.3 mL/min for 15 min. For in-line SFE experiments, the solid phase extraction cartridges (Sep-Pak C_18_ silica joined in series) were directly mounted to the SFE system effluent port to trap and to collect the bacterial RQ. After the extraction step, the collected RQ were eluted from the cartridges with pure acetone. For off-line SFE experiments, Sep-Pak C_18_ silica cartridges were not applied to SFE system effluent. Various solvents (methanol, acetone, a mixture of 10% diethyl ether and hexane) were examined as the trapping solvent. 

### 3.6. HPLC and UPLC Analysis

The chromatographic separation of quinones was performed using HPLC and UPLC. The HPLC (Shimadzu, Japan) was equipped with an ODS column (Zorbax-ODS, 4.6 mm I.D. × 250 mm, Agilent Technologies, USA) and a photo diode array detector (SPD-M10A, Shimadzu). The temperature of the column oven was maintained at 35 °C. A mixture of methanol and isopropyl ether (9:2, v/v) was used as the mobile phase at a flow rate of 1.0 mL/min. The conditions for the HPLC analysis were based on the previous study [18].

For the UPLC analysis, the improvement of performance was examined by changing the column temperature and flow rate. The influence of the flow rate (0.1, 0.2, 0.3, 0.4, 0.5 mL/min) was investigated with a column temperature of 35 °C, and the flow rate was set at 0.5 mL/min when the column temperature (25, 30, 35, 40, 45 °C) was altered. The optimal column temperature of 35 °C was used. At this column temperature and at flow rate of 0.5 mL/min, separation was achieved in 10 min with a backpressure of 8,000 psi. To select an optimal mobile phase composition, several UPLC runs with various compositions of methanol and di-isopropyl ether (90:10; 95:5; 97:3 and 100:0) as mobile phase were also performed. Considering the analysis time, methanol concentration of 100% was selected as the optimal mobile phase composition for a mixture of UQ-10 and MK-7 standards in this study.

The Waters Acquity UPLC system (Milford, MA, USA) was equipped with a binary solvent delivery manager, a sample manager and a photo-diode array detector (PDA-2996, Waters). The analytical column was a Waters Acquity UPLC™ BEH C18 column (1.7 μm, 2.1 mm × 50 mm). The mobile phase consisted of 100% methanol was pumped at a flow rate of 0.5 mL/min. Chromatography was performed at 35 ± 1 °C with a chromatographic run time of 35 min. The autosampler temperature was set at 4.0 ± 1 °C and the sample injection volume was 10 μL.

Quinone species were identified according to the retention time and the spectrum of each peak observed in the detector. The wavelengths used to quantify ubiquinones and menaquinones were 275 nm and 270 nm, respectively. The linear relationship between the logarithm of the retention times of quinones and the equivalent number of isoprenoid units (ENIU) was used to identify the quinone species [28,29,30]. The quinone contents were calculated based on their molar absorption coefficients as follows: ubiquinones, 14.4 mM^−1^cm^−1^; menaquinones, 17.4 mM^−1^cm^−1^ [31]. To enhance the objectivity of the information and to make quantitative estimates, the dissimilarity index was used. The nomenclature of the quinones was designated as follows: ubiquinones and menaquinones with n isoprene units in their side chain were abbreviated as UQ-n and MK-n, respectively. For example, UQ-8 indicates a ubiquinone with eight isoprene units and MK-8(H_2_) denotes a menaquinone with eight isoprene units where one of the double bonds is saturated with 2 hydrogen atoms [32].

## 4. Conclusions

We have investigated different methods for the determination of bacterial RQ in environmental and biological samples, namely in-line and off-line supercritical carbon dioxide extraction with solid-phase cartridge methods. The results were compared to conventional organic solvent extraction based on total amount and content of respiratory quinone. The in-line supercritical carbon dioxide extraction with the solid-phase cartridge was capable of a fast and simple determination of the microbial community population in different environmental and biological samples. The solid-phase cartridge could trap bacterial RQ from the samples in one step without further purification. Simultaneous determination of UQ and MK in the samples by UPLC was eliminated the tedious sample preparation steps. Combination of SFE and UPLC methods could be an effective tool for the characterization of the quinone profile for environment assessment and animal health management. The data obtained from this study may significantly extend information of respiratory quinone profiling. Building a comprehensive profiling database for quinone is one of the main reasons for further progression of this study.
